# Nyquist Zone Index and Chirp Rate Estimation of LFM Signal Intercepted by Nyquist Folding Receiver Based on Random Sample Consensus and Fractional Fourier Transform

**DOI:** 10.3390/s19061477

**Published:** 2019-03-26

**Authors:** Xinqun Liu, Tao Li, Xiaolei Fan, Zengping Chen

**Affiliations:** 1 National Key Laboratory of Science and Technology on ATR, National University of Defense Technology, Changsha 410073, China; liuxinqun@foxmail.com (X.L.); Xiaolei_zeno@126.com (X.F.); atrchen@sina.com (Z.C.); 2 Artificial Intelligence Research Center (AIRC), National Innovation Institute of Defense Technology (NIIDT), NO.2 Fengtai South Road, Fengtai District, Beijing 100071, China

**Keywords:** Nyquist folding receiver (NYFR), linear frequency modulated (LFM) signals, parameter estimation, random sample consensus (RANSAC), fractional Fourier transform (FrFT)

## Abstract

The Nyquist folding receiver (NYFR) can achieve a high-probability interception of an ultra-wideband (UWB) signal with fewer devices, while the output of the NYFR is converted into a hybrid modulated signal of the local oscillator (LO) and the received signal, which requires the matching parameter estimation methods. The linear frequency modulation (LFM) signal is a typical low probability of intercept (LPI) radar signal. In this paper, an estimation method of both the Nyquist Zone (NZ) index and the chirp rate for the LFM signal intercepted by NYFR was proposed. First, according to the time-frequency characteristics of the LFM signal, the accurate NZ and the rough chirp rate was estimated based on least squares (LS) and random sample consensus (RANSAC). Then, the information of the LO was removed from the hybrid modulated signal by the known NZ, and the precise chirp rate was obtained by using the fractional Fourier transform (FrFT). Moreover, a fast search method of FrFT optimal order was presented, which could obviously reduce the computational complexity. The simulation demonstrated that the proposed method could precisely estimate the parameters of the hybrid modulated output signal of the NYFR.

## 1. Introduction

Radar designers try their best to spread the energy of radar signals to multidimensional domains, including time, frequency, and space, making radar signals have a low probability of intercept (LPI) [1,2]. The reconnaissance receiver, which is designed for the LPI radar signals, should have a broad instantaneous monitoring bandwidth to keep a high probability of interception in the frequency domain and be able to process the LPI radar signals with complex signal forms. However, direct Nyquist rate sampling of the instantaneous broadband radio frequency (RF) environment is difficult because analog-to-digital converter (ADC) technology is restricted in digital bandwidth (i.e., sample rate) [3] and analog bandwidth (i.e., the ability to directly digitize high RF bands) [4]. Moreover, even though one could sample a broadband RF environment at the very accurate Nyquist rate, one would still have the problem of processing and distributing the resulting volume of data. Consequently, researchers have studied many other sampling methods or devices, such as modified Gegenbauer system [5,6], Xampling [7], and analog-to-information converters (AIC) [8,9,10]. As an efficient AIC architecture, the Nyquist folding receiver (NYFR) folds the broadband RF input signal prior to digitization by a low-speed ADC. The folding is achieved by under-sampling the RF signal with a sequence of short pulses that have a phase modulated sampling period. The RF under-sampling frequency modulation induces a Nyquist zone (NZ) dependent modulation on the received signals that allows separation and recovery of the signal information from a sparse broadband RF environment. Compared with the Gegenbauer system [5,6] and many other compress sensing (CS) based architectures [10,11] that require full signal reconstruction, the NYFR basically preserves the signal structure, and the information reconstruction can be relatively simple with conventional signal analysis methods [12].

The linear frequency modulation (LFM) signal is a typical LPI radar signal, and it has been widely used in synthetic aperture radar (SAR). LFM signal has the advantages of good concealment, wide bandwidth, low peak power, and strong anti-interference. Detection and parameter estimation of the LFM signal has been a typical issue in the radar field [13]. In the literature, there are many studies focused on the LFM signal intercepted by the conventional receiver, such as fractional Fourier transform (FrFT) [14], Radon-ambiguity transform [15], and combined Wigner–Hough transform [16,17]. Nevertheless, there are fewer methods on the NYFR, and most research on the NYFR is based on CS methods [18,19,20]. Since the NYFR output contains the added local oscillator (LO) modulation, its signal processing is more complex compared to conventional receivers.

In Reference [21], a parameter estimation algorithm based on peak search in frequency spectrum of the output signal is proposed. The parameter estimation accuracy is close to the maximum likelihood when the signal-to-noise ratio (SNR) is above −3 dB. However, the algorithm is more applicable to narrowband signals, and the performance will deteriorate along with the increase of signal bandwidth. In Reference [22], a new synchronous Nyquist folding receiver (SNYFR) structure based on composite sinusoidal frequency modulation (SFM) LO is proposed, and a parameter estimation algorithm based on instantaneous autocorrelation is presented. Effective detection of multi-component LFM signals and accurate parameters estimation are achieved when the SNR is larger than −8 dB. However, since the instantaneous autocorrelation specifies the instantaneous autocorrelation interval, this method is not suitable for short pulses which are less than a frequency modulation period of the LO. In Reference [23], an autonomous information recovery algorithm for the NYFR to determine the correct NZ of the intercepted signal is presented. A quadrature mirror filter bank (QMFB) is used to realize wavelet decomposition, and the center layer is used for signal discrimination and the modulation slope determination. By comparing the intercept modulation slope with the RF clock modulation slope, the correct NZ can be determined. Specially for SNR >−7 dB and pulse width > 1500 ns, the correct NZ can be determined with probability > 90%. However, when pulse width = 1000 ns, the probability of correct NZ determination is only about 75%, even if SNR = 10 dB.

In this paper, a parameter estimation algorithm based on random sample consensus (RANSAC) and FrFT is proposed for LFM signals intercepted by the NYFR. The paper is organized as follows: The NYFR architecture and the LFM signal intercepted by the NYFR are investigated in Section 2. The parameter estimation method for the NYFR is proposed in Section 3. The NZ estimation methods based on least squares (LS) and RANSAC are described in Section 3.1 and Section 3.2, respectively. The chirp rate estimation method based on FrFT is introduced in Section 3.3. The fast search of FrFT optimal order is presented in Section 3.4. The simulation results are given in Section 4. Section 5 summarizes the contributions of our research.

## 2. LFM Signal Intercepted by NYFR

**Figure 1:**
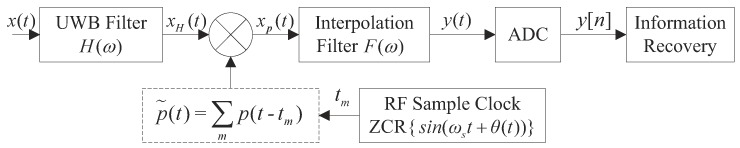
Nyquist folding receiver (NYFR) architecture.

**Figure 2:**
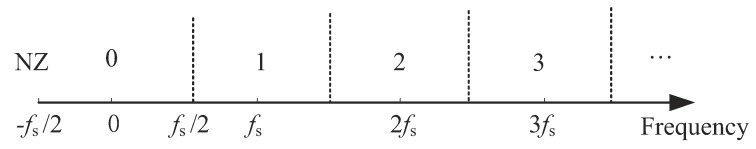
The relationship between *m* (or Nyquist Zone (NZ)) and frequency.

NYFR can achieve high-probability interception of a signal in the entire radar frequency band with a single low-speed ADC, and broadband signal acquisition can be realized without frequency sweeping in theory. Typical NYFR architecture is shown in Figure 1 [9]. Firstly, the input RF signal x(t) is filtered by an ultra-wideband (UWB) preselection filter H(ω) to filter out the uninteresting signals. Secondly, the filtered signal is mixed with a stream of time modulated short pulses $\tilde{p}\left(t\right)$, which is controlled by zero-crossing rising (ZCR) voltage time $t_{k}$ of a RF sample clock $\sin(\omega_{s}t+\theta \left(t\right))$. θ(t) is a narrowband phase modulation signal. The RF sample rate is varied continuously between $f_{s}-\Delta f$ and $f_{s}+\Delta f$. Then, after the interpolation filter, θ(t) induces a corresponding modulation mθ(t) on signals y(t), where the modulation scale factor *m* is an integer that varies with the original NZ of x(t). For the complex signal, the corresponding relationship between *m* (or NZ) and frequency is shown in Figure 2. It can be seen that *m* is a frequency-dependent signature on each component of the original signal [9]. Thirdly, y(t) is sampled to get the discrete NYFR output y[n] by ADC with sampling rate equal to the RF sample clock. Finally, y[n] is sent to digital signal processing (DSP) to get the parameters of x(t) by corresponding information recovery methods.

As can be seen from Figure 2, ($-f_{s}/2$, $f_{s}/2$) is the 0-th NZ, ($f_{s}/2$, $3f_{s}/2$) is the first NZ, ($3f_{s}/2$, $5f_{s}/2$) is the second NZ, and so on. If the SFM is used as the LO modulation type, the short pulse sequences $\tilde{p}\left(t\right)$ can be given by [21]:

(1) $$\tilde{p}\left(t\right)=\sum_{m=0}^{M-1}\exp\left\{jm[2\pi f_{s}t+m_{f}\sin\left(2\pi f_{sin}t\right)+\phi_{LO}]\right\},$$

where *m* is the *m*-th NZ, *M* is the total NZs in monitoring, $f_{s}$ is the carrier frequency of LO, $m_{f}\sin\left(2\pi f_{sin}t\right)$ is the θ(t) in RF sample clock $\sin(\omega_{s}t+\theta \left(t\right))$, $m_{f}$ is the modulation coefficient, $f_{sin}$ is the modulation frequency of SFM signal, and $\phi_{LO}$ is the initial phase of LO. It can be seen from Equation (1) that the instantaneous frequency of $\tilde{p}\left(t\right)$ is a sequence of SFM signals located at the center of their corresponding NZs. The value of NZ bandwidth is equal to $f_{s}$ when the input signal is expressed in the form of complex number.

As shown in Figure 1, the UWB filter determines the monitoring bandwidth of the NYFR. For simplicity, the output of the filter is denoted as $x_{H}\left(t\right)$. Then the output signal is mixed by the non-uniform LO to realize the folding process and the non-uniform sampled signal can be expressed as:

(2) $$x_{p}\left(t\right)=x_{H}\left(t\right)\tilde{p}\left(t\right),$$

where $x_{p}\left(t\right)$ is the input of the interpolation filter. Next, we can obtain the analog signal y(t) in the 0-th NZ from the output of the interpolation filter F(ω), and its pass band is ($-f_{s}/2$, $f_{s}/2$). Finally, the analog output of the NYFR y(t) is sampled through the ADC and the sampling rate satisfies the Nyquist sampling theorem. The original information of x(t) can be recovered in terms of y[n] and the NZ index.

**Figure 3:**
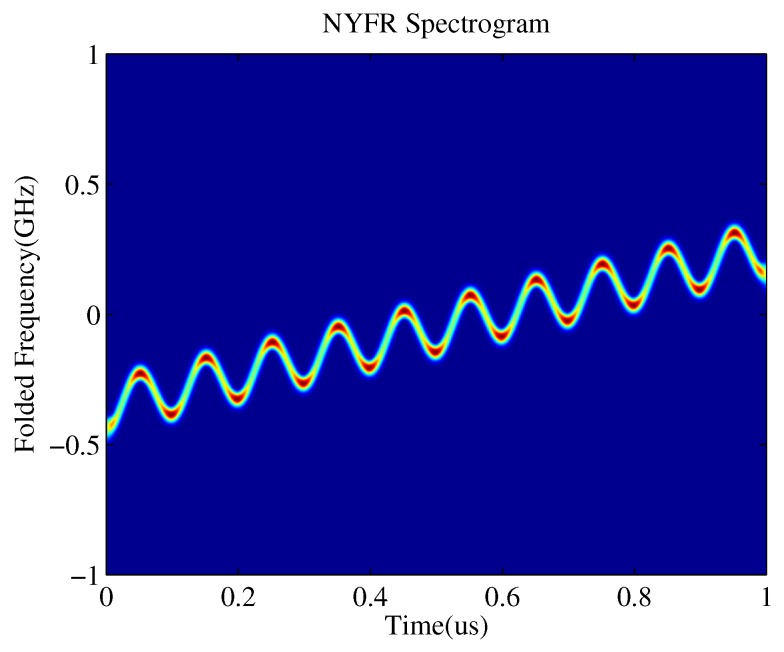
Time–frequency diagram of a linear frequency modulation (LFM) signal intercepted by NYFR.

If the input x(t) is an LFM signal, it can be normalized and formulated in Equivalent Complex Baseband (ECB) as:

(3) $$x\left(t\right)=\exp\left\{j[2\pi \left(f_{0}+0.5kt\right)t+\Phi_{0}]\right\}+\upsilon \left(t\right),$$

where $f_{0}$ is the starting frequency, *k* is the chirp rate, $\Phi_{0}$ is the initial phase, and υ(t) is the additive white Gaussian noise (AWGN) distributed in the monitoring frequency band. After mixing with the non-uniform LO, low-pass filtering, and sampling, the discrete expression of NYFR output [21] can be given by:

(4) $$y\left[n\right]=\exp\left\{j2\pi \left(f_{0}-mf_{s}\right)n+j\pi kn^{2}+j\phi_{0}-jmm_{f}\sin\left[2\pi f_{sin}n\right]-jm\phi_{LO}\right\}+\zeta \left[n\right],$$

where *m* is the NZ index indicating the original carrier frequency of the input signal, and $m=round\left(f_{0}+kt\right)/f_{s}$, round is a function that rounds the element to the nearest integer, *n* is a shorthand for $nT_{s}$, and $T_{s}$ is the sampling interval, $T_{s}=1/f_{s}$. The ζ[n] is the noise modulated by the non-uniform LO in terms of the original NZ and its power spectrum is folded into the ECB, i.e., ($-f_{s}/2,f_{s}/2$). Figure 3 illustrates an example output from a prototype NYFR for the case of an LFM input at a starting frequency of 7.65 GHz. By using the short-time Fourier transform (STFT) to obtain the time-frequency diagram, we can see that the time-frequency spectrogram of the NYFR output was an LFM/SFM hybrid modulated signal. In this paper, the mainly concerned two parameters to estimate were chirp rate (or bandwidth) and NZ. In order to facilitate the description of the principle, we assumed that the received signal just contained a single LFM and the signal frequency was in a single NZ.

## 3. Parameter Estimation of LFM Signal Intercepted by NYFR

The time–frequency ridges of a signal contain crucial information about the signal characteristics. Indeed, they mark the regions of the time–frequency plane where the signal concentrates most of its energy [24]. Therefore, an intuitive way to estimate the signal parameters on the time-frequency plane is to extract the time-frequency ridges which indicate the instantaneous frequency change of the signal. In general, each signal corresponds to a prominent ridge, which can be extracted by searching the local extreme points of the instantaneous frequency and then curve fitting. However, the traditional algorithm needs to calculate the entire time–frequency plane, which requires a large amount of computations. In order to reduce the computation complexity and achieve the real-time processing, a specific time slice can be selected to estimate the parameters. As can be seen from Figure 3, the local extreme points can be acquired at the peaks and valleys where the instantaneous frequency change rate is 0, the signal is closest to stability, and the signal bandwidth is the narrowest, but the spectrogram amplitude is higher.

**Figure 4:**
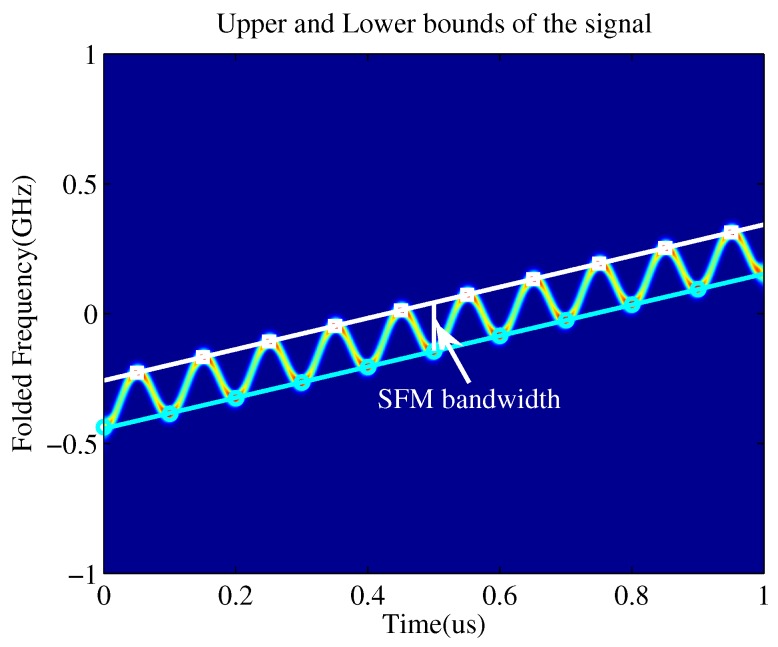
The upper and lower bounds of LFM/sinusoidal frequency modulation (SFM) hybrid modulated signal.

Assuming that the phase of the SFM LO can be accurately synchronized to the back-end DSP, these extreme points and corresponding instantaneous frequency values are used to construct “characteristic points”. Figure 4 illustrates that the upper and lower bounds of LFM/SFM hybrid modulated signal can be obtained by linear fitting in the time–frequency plane. The slope of the boundary is the estimation of the chirp rate *k*, the longitudinal interval of the upper and lower bounds corresponds to the frequency modulation bandwidth of SFM LO, which is related to the original signal NZ and can be used for NZ estimation.

As shown in Figure 4, the white boxes and cyan circles represent the “characteristic points”, while the white and cyan lines are the upper and lower bounds of the time–frequency spectrogram for the LFM/SFM hybrid modulated signal. It should be noted that the time–frequency diagram of the LFM/SFM hybrid modulated signal is not the clockwise nor counterclockwise rotation of the time–frequency diagram of the SFM signal, but it is like stretching a rectangle into a parallelogram. Therefore, the frequency modulation bandwidth of the SFM signal is the longitudinal interval of upper and lower boundaries instead of the vertical interval of two lines, which is indicated by the white longitudinal line and white arrow in Figure 4. Then, we can get the chirp rate and the starting frequency of the original LFM signal according to the slopes and intercepts of the two lines. The longitudinal interval between the two lines is the SFM modulation bandwidth.

### 3.1. NZ Index Estimation Method Based on LS

The most commonly used classical method for linear fitting is LS. Its basic principle is as follows: Assuming that the relationship between $x_{i}$ and $y_{i}$ is determined by the linear equation y=ax+b, where ($x_{i}$, $y_{i}$) is the coordinates of arbitrary point of a data set, the intercept *b* and the slope *a* are estimated parameters. Then, the overall deviation can be expressed by the sum of squared deviations, i.e., $\sum_{i=1}^{N}{\left[y_{i}-\left(ax_{i}+b\right)\right]}^{2}$. For LS, the best estimation is determined by calculating the partial derivatives of $\sum_{i=1}^{N}{\left[y_{i}-\left(ax_{i}+b\right)\right]}^{2}$ with respect to *a* and *b* and setting them to zeros:

(5) $$\frac{\partial }{\partial a}\sum_{i=1}^{N}{\left[y_{i}-\left(ax_{i}+b\right)\right]}^{2}=-2\sum_{i=1}^{N}\left(y_{i}-\hat{a}x_{i}-\hat{b}\right)=0,$$

(6) $$\frac{\partial }{\partial b}\sum_{i=1}^{N}{\left[y_{i}-\left(ax_{i}+b\right)\right]}^{2}=-2\sum_{i=1}^{N}\left(y_{i}-\hat{a}x_{i}-\hat{b}\right)=0,$$

where $\hat{a}$ and $\hat{b}$ are the estimated slope and intercept, respectively. Then the slope $\hat{a}$ can be calculated as:

(7) $$\hat{a}=\frac{\sum x_{i}^{2}\sum y_{i}-\sum x_{i}\sum x_{i}y_{i}}{N\sum x_{i}^{2}-{\left(\sum x_{i}\right)}^{2}}.$$

And the intercept $\hat{b}$ can be written as:

(8) $$\hat{b}=\frac{N\sum x_{i}y_{i}-\sum x_{i}\sum y_{i}}{N\sum x_{i}^{2}-{\left(\sum x_{i}\right)}^{2}}.$$

**Figure 5:**
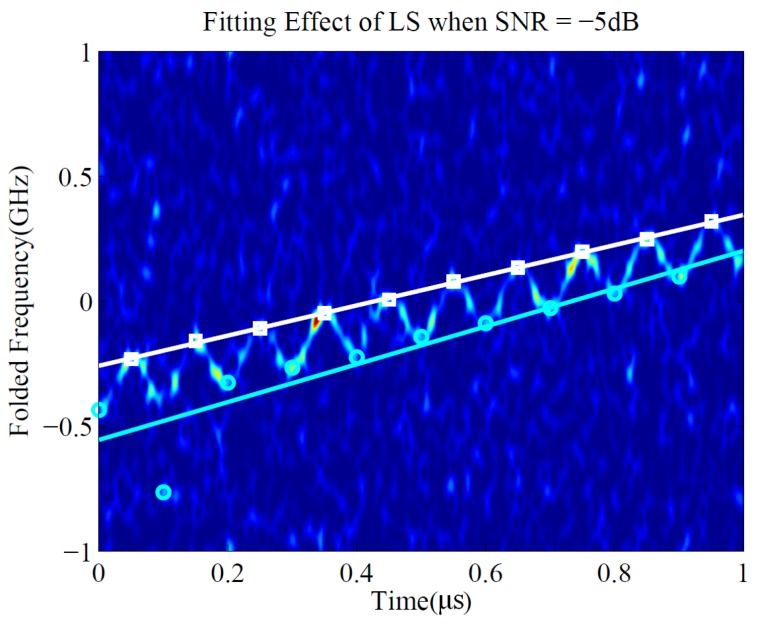
The obtained upper and lower bounds of LFM/SFM hybrid modulated signal by least squares (LS) when signal-to-noise ratio (SNR) = −5 dB.

The LS method is simple and a good parameter estimation can be obtained in most cases. However, when the SNR is lower, the individual instantaneous frequency point extracted by the maximum value in the time–frequency spectrum may deviate from the true position, and the fitting criterion of LS will cause the obtained line to seriously deviate from the true frequency-modulated line, which is depicted in Figure 5.

It can be seen that only one cyan point on the lower left of the time-frequency plane deviated from the true position. However, the deviation of the fitted straight line is severe, making LS fail to achieve a good estimation. Therefore, an iterative method based on RANSAC was proposed to improve the linear fitting effect of time-frequency characteristic points under low SNR.

### 3.2. NZ Index Estimation Method Based on Random Sample Consensus (RANSAC)

A basic assumption of the RANSAC [25] algorithm is that the data is consisted of “inliers”, i.e., data whose distribution can be explained by a set of fitting model parameters, though may be subject to noise, and “outliers” that do not fit the model. The set of inliers obtained for the fitting model is called consensus set. The idea of RANSAC is to estimate parameters of the fitting model by randomly selecting a subset of the data set. All the other data are then tested against the fitted model. The estimated model is reasonably good if a sufficient number of inliers have been found. The RANSAC algorithm iteratively repeats the above steps until the obtained consensus set in a certain iteration has enough inliers [26]. RANSAC works better than LS method because RANSAC only needs to select a set of inliers to get a good model, instead of selecting all the data points. Nevertheless, the LS can be used for line fitting after the inliers are selected.

RANSAC is accomplished with the following steps:

(1) Select a subset of the data set randomly and ensure that the size of the random samples is sufficient for determining the model parameters.
(2) Fit a model to the selected subset and then use the model to test all the other data points. If the error of a certain point is within the pre-set threshold, it is judged as an inlier; otherwise, it is an outlier. Only the model with the largest number of inliers is the best model.
(3) Repeat steps (1) and (2) for a prescribed number of iterations, the inliers corresponding to the optimal model are used to estimate the model parameters.

As for the time-frequency boundary line fitting of LFM/SFM hybrid modulated signal in this paper, since a straight line has been chosen as the fitting model, the fitting process of the upper boundary is as follows:

(1) The model is a linear equation because the boundary of the LFM/SFM hybrid modulated signal in the time–frequency plane is a line. Two sample points are randomly selected from the time–frequency “characteristic points” of the upper boundary estimated above, and a line can be determined by LS.
(2) Calculate the deviation of the remaining points from the line, i.e., the vertical distance to the line. The inliers are approximately passed by the line, whereas the outliers are far away from the line. Assuming the tolerance range is σ, find the inliers within the tolerance and count the number.
(3) Select two points randomly again and repeat steps (1) and (2). If more than 60% “characteristic points” are inliers or reaching the prescribed number of iterations, the iteration ends.
(4) Find out the iteration with the maximum number of inliers in the tolerance, and then fit all the inliers again by LS to get the straight line as the final result.

**Figure 6:**
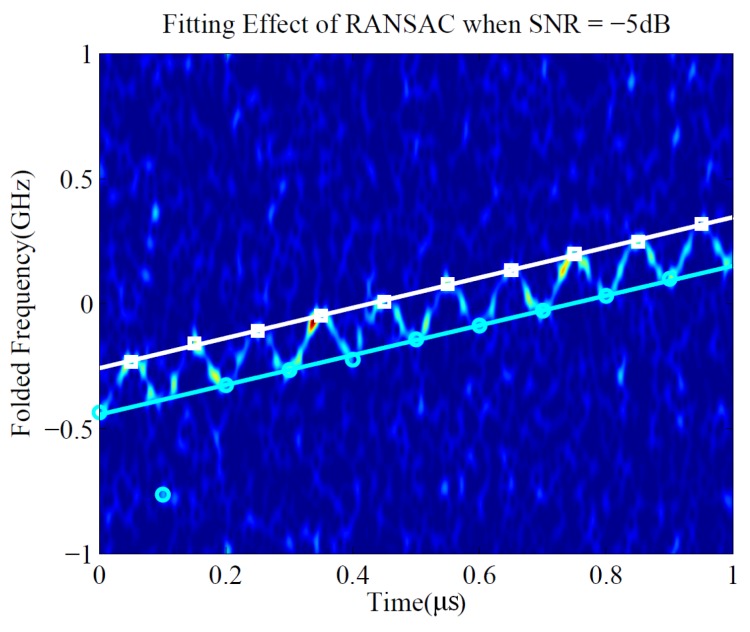
The obtained upper and lower bounds of LFM/SFM hybrid modulated signal by random sample consensus (RANSAC) when SNR = −5 dB.

The fitting process of the lower boundary is similar. Figure 6 shows the result of the RANSAC algorithm applied on the signal in Figure 5. Clearly, the RANSAC algorithm successfully avoids adapting to the outliers and only uses the inliers to estimate the model. In this way, it can avoid the contamination of outliers caused by noise near Time=0.1μs simply and effectively. The obtained linear boundary is very consistent with the real position.

**Figure 7:**
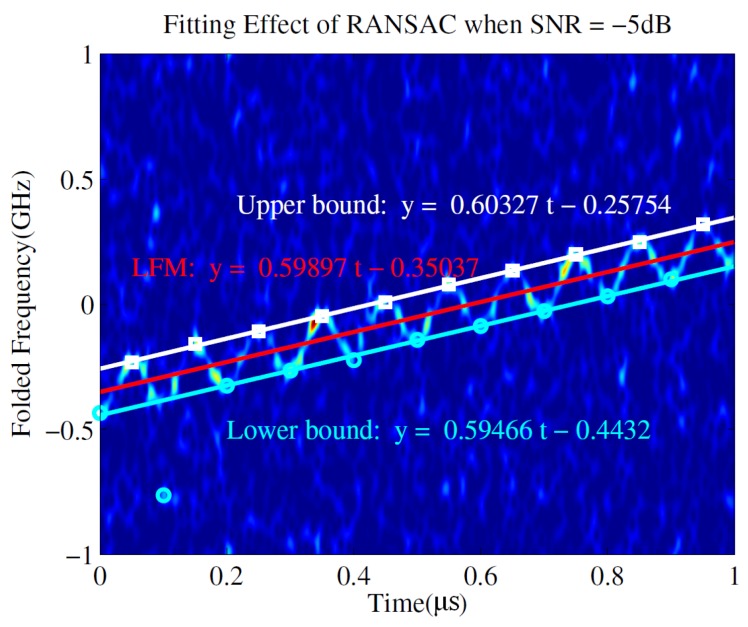
The obtained LFM time–frequency representation through upper and lower bounds when SNR =−5 dB.

To further improve the estimation accuracy, the center line of the upper and lower bounds is obtained by removing the SFM signal. Then, only the time–frequency representation of the LFM signal is preserved. The slope and intercept of the line are the chirp rate *k* and the starting frequency $f_{0}$ of the original LFM signal, respectively. The estimated time–frequency spectrogram is shown in Figure 7, where the red line is the time–frequency representation of the LFM signal obtained by the average of the other two lines (the white and cyan lines).

The longitudinal distance Δf between the upper and lower bounds is the estimated bandwidth $2mm_{f}f_{sin}$ of the SFM signal. In order to reduce the influence of linear fitting error, the longitudinal distance in the middle of the time–frequency plane can be used as Δf. Under the influence of noise inside the plane, the estimated $\hat{m}=\Delta f/\left(2m_{f}f_{sin}\right)$ is not an integer, but it is always smaller than the real value *m* and greater than m−1. So, we take the ceiling of $\hat{m}$ as an estimation of *m*. The starting frequency of the LFM signal is 7.65 GHz, which is in the 4-th NZ, and it is folded to −0.35 GHz after NYFR and the chirp rate is $6\times 10^{14}$ Hz/s. It can be seen from the figure that the intercept of the red line is −0.35037 GHz, and the estimated chirp rate is $5.9897\times 10^{14}$ Hz/s, which are both very close to the true value. The measured Δf is 0.18845 GHz, $m_{f}=2.5$, $f_{sin}=10$ MHz, and the obtained $\hat{m}=\Delta f/\left(2m_{f}m_{sin}\right)=3.7691$. The ceiling of $\hat{m}$ is 4, representing the original signal in the 4-th NZ, which is exactly the same as the actual situation.

### 3.3. Chirp Rate Estimation Method Based on FrFT

RANSAC can precisely estimate the NZ of the original signal, but the estimation accuracy of the chirp rate is a little poor. Herein, an LFM signal was obtained by removing the NZ dependent SFM in Equation (4) after correctly estimating the NZ index; then the chirp rate was estimated by the FrFT method [14]. Assume that the phase of the SFM LO could be accurately synchronized to the back-end, and then the LFM signal could be formulated as:

(9) $$y\left[n\right]=\exp\left\{j2\pi \left(f_{0}-mf_{s}\right)n+j\pi kn^{2}+j\phi_{0}\right\}+\zeta \left[n\right].$$

**Figure 8:**
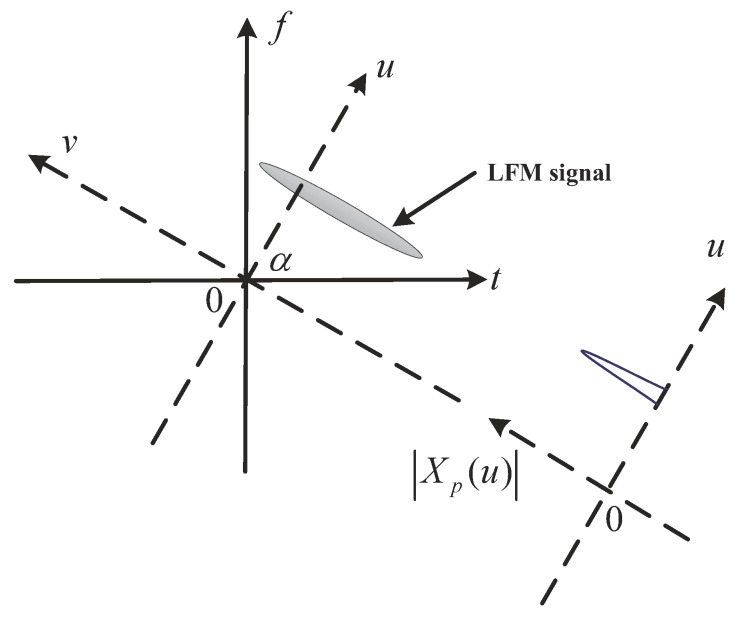
The schematic of the fractional Fourier transform (FrFT) principle.

FrFT is a kind of generalized Fourier transform, which can be interpreted as a counterclockwise rotation of the signal at any angle around the origin in the time–frequency plane. It can also be considered as the Fourier transform to the *n*-th power, where *n* can be a decimal. Thus, it can transform a signal to any intermediate domain between time and frequency. The schematic of FrFT principle is shown in Figure 8 [27].

**Figure 9:**
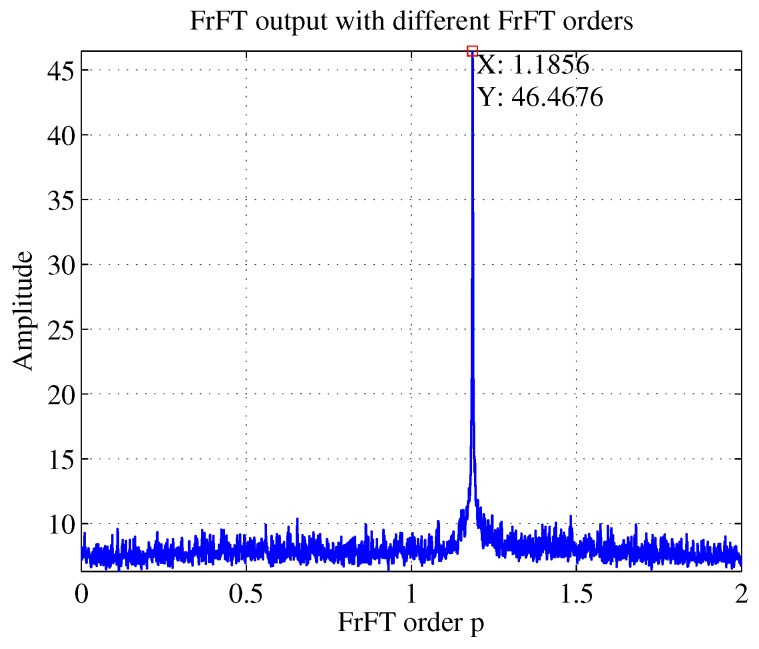
FrFT output with different FrFT orders when SNR = −5 dB.

As shown in Figure 8, for an LFM signal, if its chirp rate *k* satisfies k=−cotα, that is, when the rotation angle matches the signal chirp rate, the projection of the signal time–frequency distribution on the *u* axis will be a spike [28]. The simulation results in Figure 9 also illustrates this fact.

Recently, researchers have conducted extensive study on FrFT fast digital computation and proposed many fast implementation algorithms. The most representative one is the decomposition fast algorithm proposed in [29], where the definition of FrFT is rewritten as:

(10) $$X_{p}\left(u\right)=A_{u}\int_{-\infty }^{\infty }\left[x\left(t\right)\exp(jt^{2}\cot\left(\alpha /2\right))\right]\exp\left(-jut\csc\alpha \right)dt,\alpha \neq n\pi ,$$

where *p* is the FrFT order, $A_{u}=\sqrt{\frac{1-j\cot\alpha }{2\pi }}\exp\left(ju^{2}\cot\alpha /2\right)$ and α=pπ/2.

As can be seen in Figure 9, the abscissa of the peak is 1.1856, that was, p=1.1856. According to k=−cotα and α=pπ/2, k=0.3001 could be obtained. Because of scale transformation, inverse scaling is required to calculate the correct results after FrFT. In the simulation, $f_{s}=2$ GHz, T=1μs, so the estimated $k=0.3001f_{s}/T=6.002\times 10^{14}$ Hz/s. The estimated chirp rate by RANSAC is $5.9897\times 10^{14}$ Hz/s and the true value is $6\times 10^{14}$ Hz/s, so we could conclude that FrFT worked better than RANSAC in estimating chirp rate.

**Figure 10:**
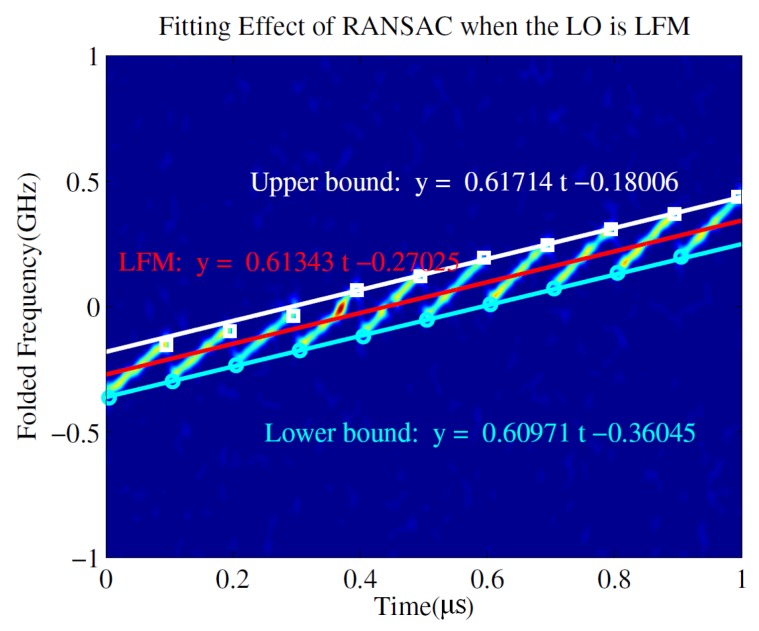
Fitting effect of random sample consensus (RANSAC) when the local oscillator (LO) is LFM.

**Figure 11:**
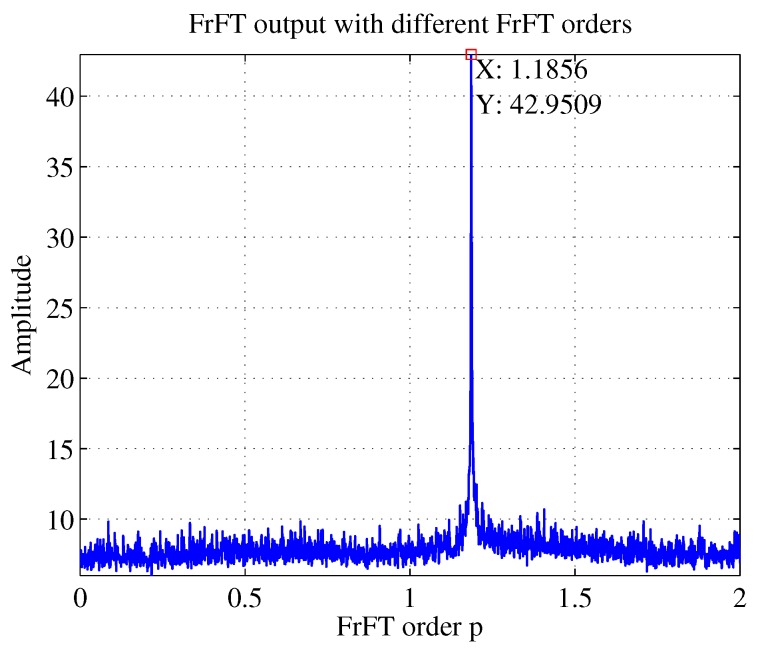
FrFT output with different FrFT orders when the LO is LFM.

The parameter estimation method based on RANSAC and FrFT was also proven to be suitable for the NYFR with a periodic LFM LO. The intercepted LFM signal can be processed similar to the above processing, and Figure 10 illustrates the time–frequency curve of an LFM signal intercepted by the NYFR with a periodic LFM LO and the fitting effect based on RANSAC when SNR = −5 dB. The rough chirp rate and starting frequency can be seen from the red line. And the more precise chirp rate is estimated by FrFT after the NZ is estimated. And the FrFT output is shown in Figure 11. The chirp rate corresponding to the peak can be calculated following by above processing.

### 3.4. Fast Search Method of FrFT Optimal Order

The premise to accurately estimate the chirp rate of LFM signal is to obtain the optimal transformation order *p*. The basic idea of conventional FrFT-based LFM signal parameter estimation is to scan the rotation angle α with equal interval. Then the FrFT of the signal is calculated with different rotation angles to form a two-dimension distribution of the signal energy on the parameter plane. In the plane, the two-dimension search of peak points is carried out according to the threshold to estimate the LFM parameters. This method requires a lot of calculations, but the accuracy of the parameter estimation is not so good.

In order to balance the estimation accuracy and computational complexity, a fast search method based on RANSAC and stepwise refinement was proposed, which did not need planar search, considerably reducing the computation cost at the same precision.

The specified steps are as follows:

(1) Set i=0, which is the approximation order and set the offset $\Delta p_{0}$, then get the rough chirp rate through RANSAC and calculate the corresponding FrFT order *p* which is denoted as $p_{0}$.
(2) i=i+1 and $\Delta p_{i}=\Delta p_{i-1}/10^{i}$, search the peak between ($p_{i-1}-\Delta p_{i-1}$) and ($p_{i-1}+\Delta p_{i-1}$) with the step $\Delta p_{i}$ and denote the *p* of the peak as $p_{i}$.
(3) Define the precision of $p_{i}$: $D=|p_{i}-p_{i-1}|$. If D≤e, *e* is the predefined threshold, $p_{i}$ is the optimal FrFT order; otherwise, go back to step (2) and continue.

For a more intuitive understanding of the computational efficiency of the fast search algorithm, we estimated the optimal order of FrFT following the above steps using a computer with an i7 Dual-Core CPU of 3.3 GHz. The approximation order *i*, estimation accuracy, and processing time are shown in Table 1.

In the above process of chirp rate estimation, the estimated chirp rate based on RANSAC was $5.9897\times 10^{14}$ Hz/s, and then the corresponding $p_{0}$ = 1.1852. It should be noted that the 0-th approximation order, i.e., the rough chirp rate was estimated by the RANSAC algorithm. Moreover, it can be seen from the table that the accuracy of parameter estimation was exponential growth with the increase of approximation orders, whereas the processing time was only linear growth. Generally, the required accuracy could be obtained at the 2nd or 3rd order.

Since the algorithm is based on peak search, the parameter estimation was not sensitive to noise when the SNR is higher. However, when the SNR was less than a certain value, the peak was submerged in the noise and the method failed. According to the simulation results, the minimum SNR applicable to this method was about −9 dB.

## 4. Simulations and Discussion

**Figure 12:**
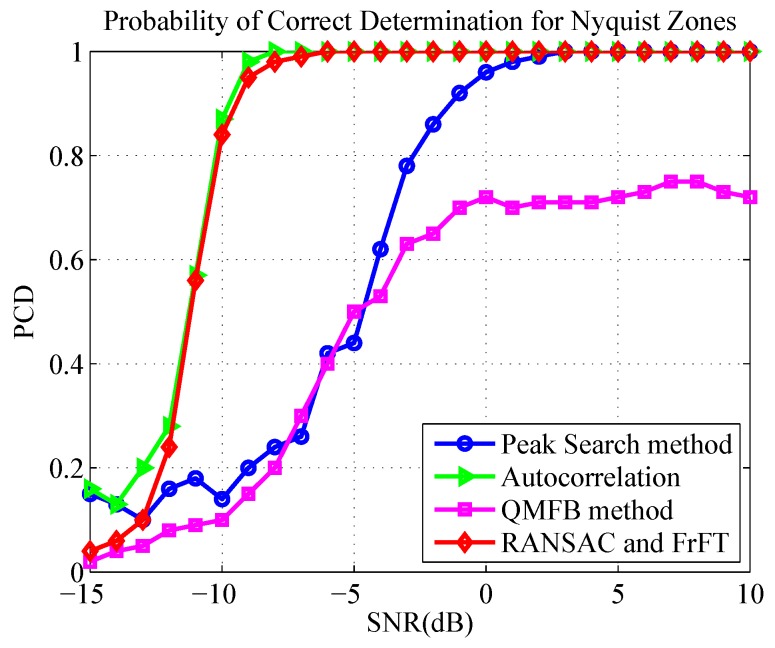
Probabilities of correct NZ determination obtained by considered methods with different SNRs.

**Figure 13:**
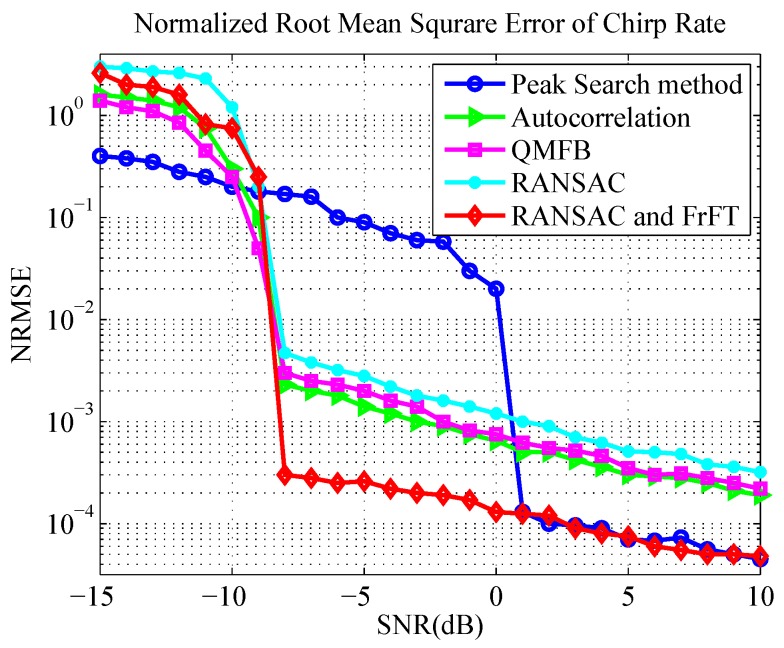
Normalized root mean square error (NRMSE) of chirp rate estimation.

To evaluate the performance of parameter estimation based on RANSAC and FrFT, we conducted Monte Carlo simulations 200 times with each SNR and calculated the probability of correct determination (PCD) for NZ and the normalized root mean square error (NRMSE) of chirp rate. The simulation parameters are listed in Table 2. We compared the proposed method with the spectrum peak search method [21], the autocorrelation method [22] and the QMFB method [23] described in Section 1. The probability of a correct NZ determination is shown in Figure 12, and the NRMSE of the chirp rate is illustrated in Figure 13.

Note that in Figure 12, the curve of RANSAC and FrFT is just contributed by RANSAC, i.e., FrFT was only used to estimate the chirp rate after the NZ was correctly estimated. The two curves (RANSAC, RANSAC and FrFT) represent two methods of chirp rate estimation in Figure 13. The difference is that the chirp rate was completely obtained by line fitting through RANSAC in the former, whereas the NZ was estimated by RANSAC first in the latter, followed by the obtainment of chirp rate using FrFT.

It is clear from the figures that the method based on RANSAC and FrFT outperformed the other methods, and the estimation performance of the chirp rate was outstanding and stable when SNR ≥−8 dB. The correct ratio of NZ index estimation was greater than 90% when SNR ≥−9 dB and reached 100% when SNR ≥−7 dB. This resulted from the fact that the method took full advantages of RANSAC and FrFT, i.e., RANSAC could accurately determine the NZ where the signal was located, and FrFT could precisely estimate the chirp rate of the signal.

In contrast, we can see that RANSAC method alone and instantaneous autocorrelation method have a similar performance in NZ determination, whereas the performance of instantaneous autocorrelation is slightly better than that of RANSAC in NRMSE of chirp rate. The main reason is that the RANSAC method fails to use the information of all sampling points. Moreover, the frequency resolution of RANSAC is limited by the length of the STFT window, which affects the accuracy of the instantaneous frequency of the signal. However, the autocorrelation method requires specific autocorrelation interval, which is not applicable to the signal less than an LO frequency modulation period. That is, the autocorrelation is interval-dependent, but the method of RANSAC is not.

The spectrum peak search method has a requirement for high SNR, and NZ can be correctly estimated only when SNR > 1 dB. The reason is that the spectral peak search method is applicable to narrowband signals, such as a single carrier signal intercepted by NYFR. Since the frequency aggregation of wideband modulated signals is not as good as the single carrier frequency signal, the spectrum peak may not be the highest after the SFM is completely removed from the hybrid modulated signal. This implies that the spectrum peak search method is not very suitable for analysis of a broadband signal intercepted by NYFR. It should be noted that the estimation of *k* can achieve higher accuracy when SNR ≥1 dB, and this is because FrFT is used to estimate the chirp rate after correct estimation of NZ.

In the QMFB method, only the center layer is used for signal discrimination and the chirp rate determination. Since it does not make full use of all the sampling data, its performance is as expected. It can be seen from Figure 12, for pulse width 1000 ns, the PCD is only about 75%, even if SNR reaches 10 dB.

## 5. Conclusions

The NYFR is an undersampling receiver that can intercept signals in the entire frequency band with a single ADC and without frequency sweeping in theory. In this paper, an intuitive and simple parameter estimation method based on RANSAC and FrFT for NYFR was proposed. First, we used STFT to obtain the time–frequency spectrogram of the SFM/LFM hybrid modulated signal. Then we had to get the NZ where the signal was located with LS and RANSAC. Finally, the chirp rate of LFM signal was obtained through FrFT. The simulation results showed the validity and reliability of the proposed method. For ease of deduction, we assumed that the received signal just contained a single LFM signal, and the frequency range of the LFM signal did not cross the NZ junctions. Therefore, the processing of multiple signals in NYFR should be studied further.

## Figures and Tables

**Table 1:** The approximation order, estimation accuracy, and time cost.

Approximation Order	Estimated Chirp Rate	Estimation Error	Processed Time
First order	$5.98987\times 10^{14}$ Hz/s	$1.013\times 10^{12}$ Hz/s	0.4216 s
Second order	$6.002\times 10^{14}$ Hz/s	$2\times 10^{11}$ Hz/s	4.068 s

**Table 2:** The simulation settings.

Name	Variable	Value
Monitoring frequency band	*f*	1–21 GHz
Amount of NZ	*M*	10
Average sampling rate	$f_{s}$	2 GHz
LO modulation coefficient	$m_{f}$	2.5
Sinusoidal modulation frequency	$f_{sin}$	10 MHz
Signal length	*T*	1 μs
Simulation points	*N*	2000 points
Signal amplitude	*A*	1
Chirp rate	*m*	600 MHz/μs
Start frequency	$f_{0}$	7.65 GHz
Initial phase of LO	$\phi_{LOS}$	0
Initial phase of LFM	$\phi_{0}$	0
NZ index	m	4
